# Blood volume deficit in postural orthostatic tachycardia syndrome assessed by semiautomated carbon monoxide rebreathing

**DOI:** 10.1007/s10286-024-01091-8

**Published:** 2024-11-30

**Authors:** Surat Kulapatana, Vasile Urechie, Stefano Rigo, Abigail Mohr, Yuliya A. Vance, Luis E. Okamoto, Alfredo Gamboa, Cyndya Shibao, Italo Biaggioni, Raffaello Furlan, André Diedrich

**Affiliations:** 1 Autonomic Dysfunction Center, Department of Medicine, Division of Clinical Pharmacology, Vanderbilt University Medical Center, 1161 21 Avenue South, Suite S3116 MCN, Nashville, TN 37232‑2600 USA; 2 Department of Biomedical Engineering, Vanderbilt University, Nashville, TN USA; 3 Tulane University School of Medicine, New Orleans, USA; 4 Humanitas Clinical and Research Center- IRCCS, Via Alessandro Manzoni, 56 Rozzano, Italy; 5 Department of Biomedical Sciences, Humanitas University, Pieve Emanuele, Italy; 6 Department of Physiology, Faculty of Medicine Siriraj Hospital, Mahidol University, Bangkok, 10700 Thailand; 7 Clinical Research Unit, myDoctorAngel Sagl, Bioggio, Switzerland

**Keywords:** Blood volume, Plasma volume, Red blood cell volume, Carbon monoxide rebreathing, Postural orthostatic tachycardia syndrome

## Abstract

**Purpose:**

The semiautomated carbon monoxide (CO) rebreathing method has been introduced as a noninvasive and radiation-free blood volume estimation method. We tested whether the semiautomated CO rebreathing method can detect the blood volume deficit in postural orthostatic tachycardia syndrome (POTS). In addition, we explored the relationship between blood volume estimated from CO rebreathing and body impedance.

**Patients and methods:**

We recruited 53 subjects (21 female patients with POTS, 19 healthy female participants, and 13 healthy male participants) to record blood volumes and hemodynamic data. Blood volumes were measured by CO rebreathing and segmental body impedance. Linear regression models to predict normal values of red blood cell volume (RBCV), plasma volume (PV), and total blood volume (BV) were developed. Percentage deviations from the predicted normal volumes were calculated.

**Results:**

Patients with POTS had lower RBCV (25.18 ± 3.95 versus 28.57 ± 3.68 mL/kg, *p* = 0.008, patients with POTS versus healthy female participants), BV (64.53 ± 10.02 versus 76.78 ± 10.00 mL/kg, *p* < 0.001), and BV deviation (−13.92 ± 10.38% versus −0.02 ± 10.18%, *p* < 0.001). Patients with POTS had higher supine heart rate (HR) (84 ± 14 versus 69 ± 11 bpm, *p* < 0.001) and upright HR (123 ± 23 versus 89 ± 22 bpm, *p* < 0.001). We found a correlation between BV deviation and upright HR in patients with POTS (*r* = −0.608, *p* = 0.003), but not in healthy participants. Volumes from the CO rebreathing and body impedance were well correlated (*r* = 0.629, *p* < 0.001).

**Conclusions:**

The CO rebreathing method can detect BV deficit, as well as the RBCV deficit in patients with POTS. The negative correlation between BV deviation and upright HR indicates that hypovolemia is one of the pathophysiological causes of POTS. Correlations between body impedance and CO rebreathing volume suggest its usefulness for measurements of volume changes.

## Introduction

Postural orthostatic tachycardia syndrome (POTS) is a burdensome autonomic disorder affecting approximately 1% of the US population. Its main clinical feature is an excessive heart rate increase upon standing, associated with orthostatic symptoms such as lightheadedness, nausea, and headache [1–3]. The pathophysiology of POTS is heterogeneous and often overlapping. Studies have shown that most patients with POTS have hypovolemia [1, 4, 5]. Low blood volume could lead to compensatory orthostatic tachycardia to maintain cardiac output and blood pressure via baroreflex mechanisms. It is therefore important to know the blood volume status of patients with POTS to better understand the disease and to individualize therapies (e.g., to increase sodium and water intake and use of fludrocortisone) [1, 2, 6, 7].

Absolute assessments of blood volume are based on indicator or dye-dilution methods. The basic principle is that, if a known amount of indicator (*A*) is injected into a system and the concentration (*C*) of the indicator can be measured, then one can solve for the unknown volume (*V*), such as plasma volume, using the formula *V* = *A*/*C* [8]. Evans Blue has been widely used as a dye for blood volume determination but is no longer recommended because of probable toxicity and carcinogenic effects [9]. Indocyanine green is another dye, but its excretion rate is limited by liver function [10]. Arguably, the gold standard of blood volume measurement uses radiolabeled iodinated human serum albumin (I-HSA) [11] or chromium isotope-labeled red blood cells (^51^Cr-RBC) [12], but this have the drawback of exposure to radioactivity.

Rebreathing small amounts of carbon monoxide (CO) offers a noninvasive, safe alternative to measure hemoglobin mass and blood volume. The method consists of inhaling a bolus amount of CO and subsequently measuring carboxyhemoglobin blood content. However, the manual CO rebreathing procedure can be complicated and prone to inaccuracies [13, 14], with a total measurement error of 4–5% [5]. Recently, it was shown that, with improved sensor technologies and semiautomated device support, the total error measurement can be reduced to 0.8–1.6% (Detalo Device Specifications, Detalo Performance, Detalo Health, Hørsholm, Denmark), approaching the precision of the gold-standard, radioactive labeling method [16]. This new method has been used successfully to estimate blood volume in healthy subjects [16, 17]. To evaluate whether the semiautomated CO rebreathing method can detect red blood cell, plasma, and blood volume deficits in patients with POTS, we compared these measurements between patients with POTS and healthy controls. Additionally, we assessed volume by a noninvasive segmental body impedance method. The fluid volume in each body region can be estimated from the impedance calculated from voltage changes in response to an injected electrical current [18–21]. We explored the relationship between CO rebreathing estimated volumes and resting hemodynamics, as well as the correspondence with volumes from segmental body impedance in both patients with POTS and healthy controls.

## Protocol

We enrolled 53 subjects (21 female patients with POTS,19 healthy female participants, and 13 healthy male participants) at the Vanderbilt Autonomic Dysfunction Center between 2020 and 2023. The inclusion criteria for patients with POTS were chronic orthostatic symptoms, heart rate increase of at least 30 bpm upon 10 min of standing, and no orthostatic hypotension. Exclusion criteria were any disabling cardiovascular disease, other conditions known to cause postural tachycardia or low blood volume, and a history of coronavirus disease 2019 (COVID-19) infection.

The study was approved by the Vanderbilt University Institutional Review Board in Human Research. The protocol was part of a registered clinical trial (NCT04050410). All participants gave written consent.

All patients stopped medications affecting heart rate, blood pressure, blood volume, and autonomic nervous system for at least five half-lives before studies. After resting in supine position for ≥ 30 min to allow full-body fluid redistribution, subjects underwent CO rebreathing for 10 min with pre- and post-rebreathing blood draws. Electrocardiogram (Ivy 450C, Ivy Biomedical Systems, Inc, Brandford CT, USA), continuous finger blood pressure (Finapres NOVA, the Netherlands), contralateral intermittent brachial blood pressure (Ivy 450 C, Ivy Biomedical Systems, Inc, Brandford CT, USA), and segmental body impedance (BIM, Diefenbach GmbH, Germany) were also recorded during a 5-min resting period in supine position. Hemodynamic parameters including stroke volume, cardiac output, and total peripheral resistance were automatically derived from Finapres’s software (Modelflow^®^, Finapres Medical System BV, the Netherlands) [22] and averaged over the 5-min resting period by using our customized MATLAB software (Physiowave^©^, A. Diedrich, Vanderbilt University Medical Center, TN, USA). Upright heart rate (HR) and blood pressure (BP) were obtained during the 10-min active stand test at 3, 5, and 10 min. The maximal upright HR and corresponding BP were selected.

### Carbon monoxide rebreathing

A semiautomated blood volume analyzer (Detalo Performance, Detalo Health, Hørsholm, Denmark) was used to determine hemoglobin mass. The device can precisely deliver a bolus of chemically pure 99.997% CO (UN1016, A-L Compressed Gases, Nashville, USA) to a closed rebreathing circuit and analyze residual gas status after the rebreathing. The reported total error of the Detalo device is between 0.8% and 1.6%.

After 30 min of resting in supine position, a “pre-test” blood sample was drawn for the carboxyhemoglobin fraction (%HbCO), hemoglobin (Hb) concentration, and hematocrit (Hct). Then, subjects were connected to the Detalo device through a mouthpiece. Breathing 100% oxygen (O_2_) was started, and a nose clip was put on. After breathing comfortably for 1 min, CO at a dose of 1 mL/kg was injected into the circuit at the end of inhalation. Subjects rebreathed for 10 min to allow all the CO to be inhaled, evenly distributed, and bound with hemoglobin to form carboxyhemoglobin (HbCO). During rebreathing, a fresh gas flow with 100% O_2_ from oxygen tanks was provided to avoid gas depletion, and a soda-lime adsorbent was used to avoid carbon dioxide (CO_2_) buildup. After 10 min, blood was drawn for a “post-test” sample. Then, subjects were asked to exhale to completely empty their lungs before disconnecting the mouthpiece. Lastly, the CO left in the circuit was measured using a handheld analyzer (Monoxor^®^ Plus, PA, USA).

Hb and carboxyhemoglobin concentrations (%HbCO) were measured in triplicate with an ABL80 SC80 OSM gas analyzer (Radiometer America Inc, Brea, CA, USA). Hematocrit was measured in triplicate with a high-precision Ultrakit hematocrit device (EKF USA, Boerne, TX, USA).

Total hemoglobin mass (Hb_mass_) can be computed from Eq. 1 [14]. The formula is based on the fact that carboxyhemoglobin is proportional to the absorbed CO with a factor of ¼, since 1 mol of hemoglobin can firmly bind to 4 mol of CO. We transformed CO volume to moles following the ideal gas law. The %HbCO change was derived from the pre- and post-blood draws. The absorbed CO was determined as the difference between the injected CO (1 mL/kg) and the CO remaining in the circuit at the end of the rebreathing.

Equation 1 enables the calculation of hemoglobin mass by the CO rebreathing technique using the dilution principle:

1 $$\mathrm{Hb}_\mathrm{mass}=\left(\frac{\frac{\left({V}_\mathrm{CO start}-{V}_\mathrm{CO left}\right) x \frac{{P}_\mathrm{atm}}{R \times T}}{4}}{\mathrm{\%HbCO}_\mathrm{post}-\mathrm{\%HbCO}_\mathrm{pre}}\right) \times 100 \times \mathrm{Hb}_\mathrm{mw} ,$$

where *V*_CO start_ is the injected CO volume (L), *V*_CO left_ is the CO volume remaining in the circuit at the end of rebreathing (L), *P*_atm_ is atmospheric pressure (atm), *T* is temperature (K), *R* is the ideal gas constant (0.08206 L atm/mol/K), %HbCO_post_ is the fraction of carboxyhemoglobin after 10 min of CO rebreathing (%), %HbCO_pre_ is the fraction of carboxyhemoglobin before 10 min of CO rebreathing (%), Hb_mw_ is the molecular weight of hemoglobin (6.44 × 10^4^ g/mol), and Hb_mass_ is the mass of hemoglobin (g).

After Hb_mass_ was determined, the blood volume was derived from Hb_mass_ and Hb concentration using Eq. 2. Once the blood volume was determined, the plasma volume and red blood cell volume were calculated as fractions of blood volume, using Hct.

Equation 2 enables the blood volume, plasma volume, and red blood cell volume calculations:

2a $$BV= \frac{\mathrm{Hb}_\mathrm{mass}}{\left[\mathrm{Hb}\right]},$$

2b $$\mathrm{RBCV} = \frac{\mathrm{Hct}}{100} \times \mathrm{BV},$$

2c $$\mathrm{PV}= \left(1-\frac{\mathrm{Hct}}{100}\right) \times \mathrm{BV},$$

where BV is the blood volume (L), RBCV is the red blood cell volume (L), PV is the plasma volume (L), Hb_mass_ is the hemoglobin mass (g), [Hb] is the hemoglobin concentration (g/L), and Hct is the hematocrit (%).

### Blood volume prediction

The default predicted blood volume formula provided in the Detalo device software is an equation proposed by Nadler et al. (Eq. 3) [23, 24]. Additionally, we performed a linear regression of our healthy control volume data with weight, height, and sex to estimate predicted “normal” blood volume values.

We compared the blood volume measured using our CO rebreathing values with the predicted blood volume from each method and report the percentage difference from the predicted blood volume (BV_deviation_) according to Eq. 4.

Nadler’s predicted blood volume equations are

3a $$\mathrm{BV}_\mathrm{male}\left(\mathrm{L}\right)=0.3669{ \times \mathrm{height}\left(\mathrm{m}\right)}^{3}+0.03219 \times \mathrm{weight}\left(\mathrm{kg}\right)+0.6041,$$

3b $$\mathrm{BV}_\mathrm{female}\left(\mathrm{L}\right)= 0.3561 \times {\mathrm{height}\left(\mathrm{m}\right)}^{3}+0.03308 \times \mathrm{weight}\left(\mathrm{kg}\right)+0.1833.$$

The percentage difference from the predicted blood volume is calculated as

$$\mathrm{BV}_\mathrm{deviation}(\%)=100 \times \frac{\mathrm{BV}_\mathrm{measured}-\mathrm{BV}_\mathrm{predicted}}{\mathrm{BV}_\mathrm{predicted}},$$

where BV_deviation_ is the percentage difference from the predicted blood volume (%), BV_measured_ is the blood volume measured from the CO rebreathing method (L), and BV_predicted_ is the predicted blood volume from either Nadler’s equation or control linear-fit equation (L).

### Segmental body impedance

**Fig. 1:**
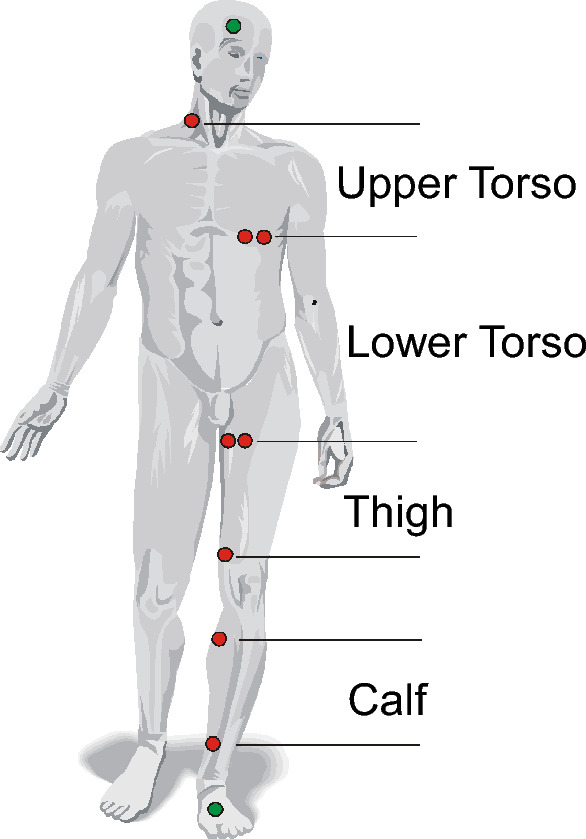
Electrode positions for segmental body impedance measurements; green, current-injection electrode; red, voltage-sensing electrodes

Ten Ag/AgCl gel electrodes (Ambu Blue Sensor VL, AmbuUSA, Columbia MD) were placed along the body axis. The selected skin areas were cleaned with alcohol. Two current-delivery electrodes were attached on the right frontal area of the head and dorsal area of left foot. Voltage-sensing electrodes were placed on the center of the right supraclavicular fossa, the left anterior axillary line at the level of the xiphoid, the left groin, the medial side of the left thigh at 5 cm above the knee, the medial side of the left calf at 5 cm below the knee, and at 5 cm above the left medial malleolus (Fig. 1). The distance between each sensing electrode was recorded. A battery-powered body impedance measurement device (BIM, Diefenbach GmbH, Germany) applied a small alternating current (0.6 mA at 32.7 kHz) to record the impedance of the thorax, abdomen, thigh, and calf regions simultaneously for 5 min in supine resting position.

Plasma volume is inversely correlated with the impedance and can be estimated by using Eq. 5 [20], with customized MATLAB software (Physiowave^©^, A. Diedrich, Vanderbilt University Medical Center, TN, USA). Once each segmental fluid volume was obtained, the total volume can be computed from the body impedance as the summation of the thoracic volume, abdominal volume, two times the thigh volume, and two times the calf volume.

The volume was calculated from the segmental body impedance as

$$V=\frac{{L}^{2} \times {p}_\mathrm{eff}}{R} \times 1000,$$

where *V* is the segmental volume (L), *L* is the segmental length (m), *p*_eff_ is the effective resistance of plasma (1.0 Ω m), and *R* is the segmental impedance (Ω).

### Statistical analysis

Results are presented as mean ± standard deviation (SD). The mean of the continuous hemodynamic values was computed from the beat-to-beat heart rate (HR), systolic blood pressure (SBP), and diastolic blood pressure (DBP) determined by using our customized MATLAB software (Physiowave^©^, A. Diedrich, Vanderbilt University Medical Center, TN, USA). Data normality was checked by the Anderson–Darling test using MATLAB software. Data were compared separately between two groups (healthy male participants versus healthy female participants, and healthy female participants versus female patients with POTS) by using the Wilcoxon rank-sum test if the data were not normally distributed, while the unpaired -test was used if they had normal distribution. Correlations between the blood volume calculated by using the rebreathing and the impedance methods were analyzed by Pearson’s linear correlation. Correlations between BV_deviation_ and baseline hemodynamic data, i.e., heart rate and blood pressure, were also analyzed. All statistical analyses were performed in MATLAB. *p*-Value ≤ 0.05 was considered to indicate statistical significance.

## Results

### General characteristics

We enrolled 21 female patients with POTS, 19 healthy female participants, and 13 healthy male participants. There were no significant differences in age, weight, and height between the female patients with POTS and the healthy female participants (Table 1). As expected, the baseline heart rate in patients with POTS was significantly higher than in controls.

**Table 1:** General characteristics and baseline hemodynamic values in healthy participants and in patients with postural orthostatic tachycardia syndrome (POTS)

	Healthy male participants (*n* = 13)	Healthy female participants (*n* = 19)	Female patients with POTS (*n* = 21)
Age (years)	33 ± 10	32 ± 10	28 ± 7
Weight (kg)	83 ± 13^#^	64 ± 8	68 ± 11
Height (cm)	177 ± 6^#^	166 ± 7	168 ± 7
**Supine**			
Heart rate (beats/min)	70 ± 11	69 ± 11	84 ± 14^*^
Systolic blood pressure (mmHg)	117 ± 7^#^	102 ± 9	111 ± 11^*^
Diastolic blood pressure (mmHg)	71 ± 8	67 ± 6	71 ± 7
Stroke volume (mL)	100 ± 10^#^	83 ± 18	87 ± 13
Cardiac output (L/min)	7.2 ± 1.4^#^	5.7 ± 1.5	7.3 ± 1.4^*^
Total peripheral resistance (dyn s/cm^5^)	1017 ± 204	1230 ± 388	980 ± 178^*^
**Upright**			
Heart rate (beats/min)	92 ± 11	89 ± 22	123 ± 23^*^
Systolic blood pressure (mmHg)	117 ± 7	109 ± 16	111 ± 22
Diastolic blood pressure (mmHg)	78 ± 5	81 ± 17	85 ± 14

Values presented as mean ± SD. * *p* < 0.05 between healthy female participants and female patients with POTS; # *p* < 0.05 between healthy male and healthy female participants

### Blood volume assessments

**Fig. 2:**
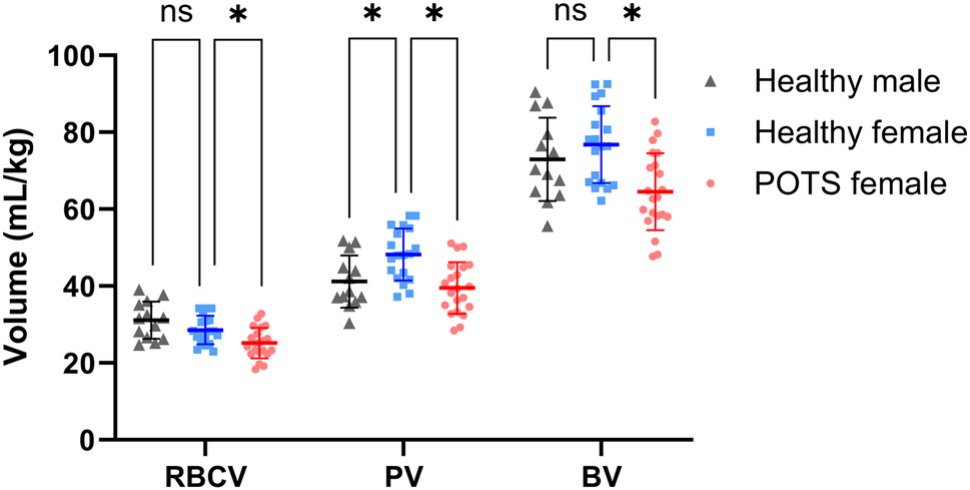
Red blood cell volume (RBCV), plasma volume (PV), and blood volume (BV) estimated by the carbon monoxide rebreathing method in healthy participants and in patients with postural orthostatic tachycardia syndrome (POTS); *p < 0.05; ns, not significant

Healthy male participants had higher hematocrit and higher hemoglobin concentration than healthy female participants. Comparing within the female sex, blood analyses showed higher Hct (38.94 ± 2.26% versus 37.28 ± 1.99%, *p* = 0.018) in patients with POTS, but lower Hb_mass_ (8.54 ± 1.51 versus 9.51 ± 1.33 g/kg, *p* = 0.039) and RBCV (25.18 ± 3.95 versus 28.57 ± 3.68 mL/kg, *p* = 0.008) (Table 2 Fig. 2). Patients with POTS also had significantly lower plasma volume (39.50 ± 6.71 versus 48.20 ± 6.73 mL/kg, *p* < 0.001) and blood volume (64.53 ± 10.02 versus 76.78 ± 10.00 mL/kg, *p* < 0.001) than healthy female participants (Table 2; Fig. 2).

**Table 2:** Red blood cell, plasma, and blood volume determined by the carbon monoxide rebreathing method in healthy participants and patients with postural orthostatic tachycardia syndrome (POTS)

	Healthy male participants (*n* = 13)	Healthy female participants (*n* = 19)	Female patients with POTS (*n* = 21)
Hematocrit (Hct, %)	43.42 ± 2.84^#^	37.28 ± 1.99	38.94 ± 2.26^*^
Hemoglobin concentration (Hb, g/dL)	14.84 ± 0.99^#^	12.41 ± 1.01	13.06 ± 0.74^*^
Hemoglobin mass (Hb_mass_, g/kg)	10.77 ± 1.55^#^	9.51 ± 1.33	8.54 ± 1.51^*^
Red blood cell volume (RBCV, mL/kg)	31.13 ± 4.85	28.57 ± 3.68	25.18 ± 3.95^*^
Plasma volume (PV, mL/kg)	41.18 ± 6.81^#^	48.20 ± 6.73	39.50 ± 6.71^*^
Blood volume (BV, mL/kg)	72.97 ± 10.85	76.78 ± 10.00	64.53 ± 10.02^*^

Values presented as mean ± SD. * *p* < 0.05 between healthy female participants and female patients with POTS; # *p* < 0.05 between healthy male and healthy female participants

### Estimate of blood volume deficits from predicted normal values

**Fig. 3:**
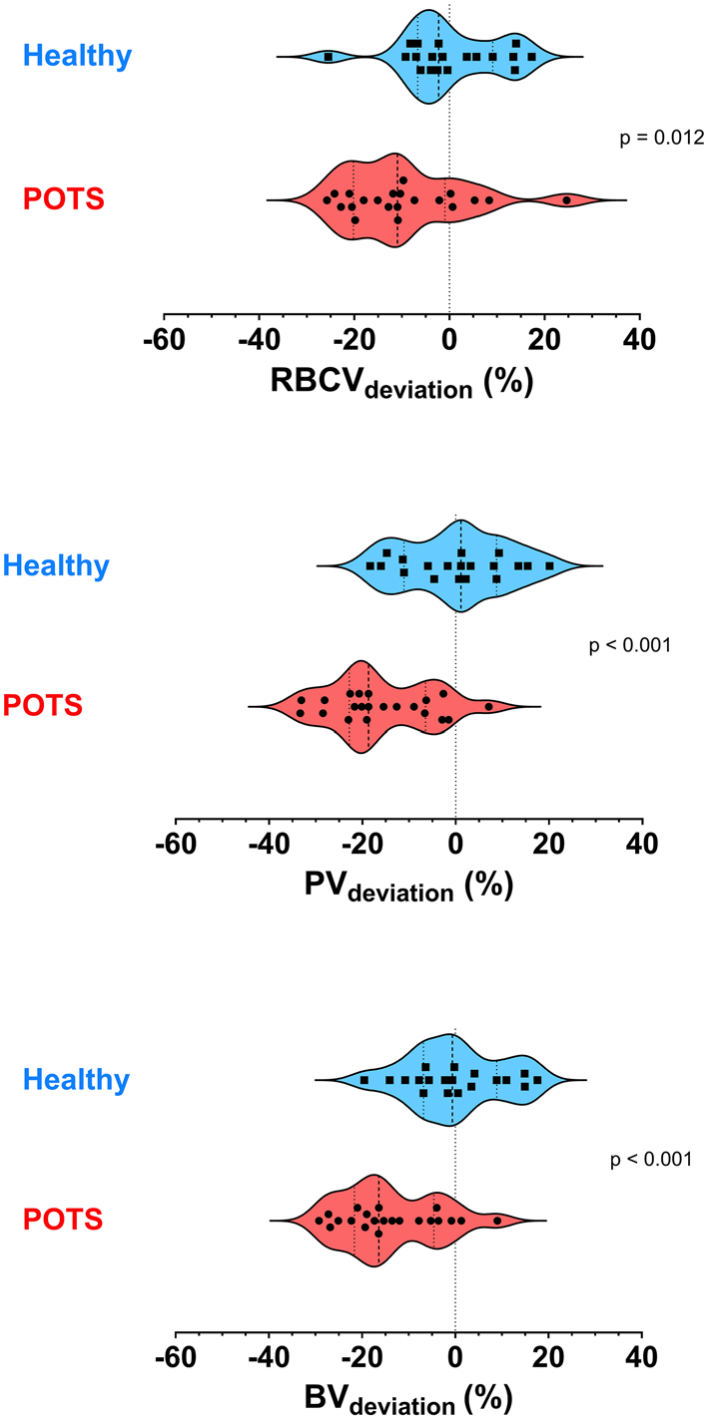
Volume deviation of red blood cell volume (RBCV_deviation_, top), plasma volume (PV_deviation_, middle), and blood volume (BV_deviation_, bottom) by carbon monoxide rebreathing in healthy female participants and female patients with postural orthostatic tachycardia syndrome (POTS)

We applied linear regression models on healthy control data of RBCV, PV, and BV to estimate predicted normal volumes based on weight, height, and sex. Estimated linear regression equations are presented in Table 3. The percentage differences, calculated from the predicted volume and measured volume, showed that female patients with POTS had reduced red blood cell volume (−9.72 ± 12.45% versus −0.01 ± 10.45%, *p* = 0.012, versus healthy female participants), plasma volume (−16.08 ± 10.99% versus −0.02 ± 11.04%, *p* < 0.001), and blood volume (−13.92 ± 10.38% versus −0.02 ± 10.18%, *p* < 0.001) (Fig. 3). In contrast, the percentage differences using predicted blood volumes calculated by Nadler’s formula were +13.29 ± 15.37% for healthy male participants, +23.88 ± 13.13% for healthy female participants, and +5.61 ± 13.57% in patients with POTS.

**Table 3:** Linear regression models for prediction of normal volumes corrected by weight and height

*Y*	*a*	*b*	*c*	Adjusted *R* Squared - Value	*p*-Value
RBCV, female participants	0.01125	1.2369	−0.96024	0.321	0.018
RBCV, male participants	0.023329	1.5225	−2.0524	0.344	0.049
PV, female participants	0.014049	3.1442	−3.0639	0.325	0.017
PV, male participants	0.023897	7.4107	−11.652	0.678	0.001
BV, female participants	0.025309	4.3797	−4.0222	0.354	0.012
BV, male participants	0.042912	9.8666	−14.944	0.577	0.006

Linear regression for variables *Y* = *a* × *W* + *b* × *H* + *c*, where *Y* is volume in liters, *W* is weight in kg, and *H* is height in meters. RBCV red blood cell volume, PV plasma volume, BV blood volume

### Correlation analysis between blood volume and hemodynamics

**Fig. 4:**
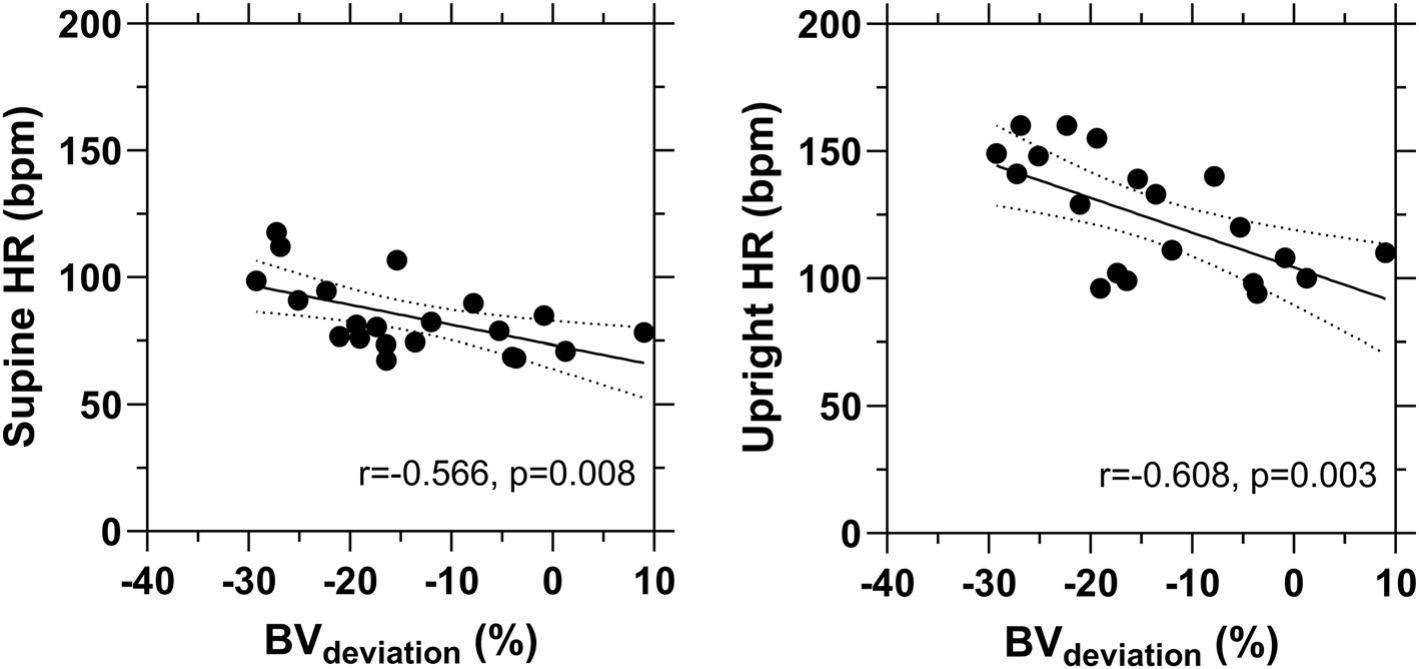
Correlation analyses between blood volume deviation (BV_deviation_) and supine heart rate (left) and upright heart rate (right) in female patients with postural orthostatic tachycardia syndrome (POTS)

CO rebreathing blood volume status (percentage difference from predicted blood volume by the regression model, BV_deviation_) was negatively correlated with resting heart rate, in both supine (*r* = −0.566, *p* = 0.008) and upright (*r* = −0.608, *p* = 0.003) positions (Table 4; Fig. 4), and systolic blood pressure (*r* = −0.563, *p* = 0.008) in participants with POTS. There were no significant correlations between volume status and baseline hemodynamic data in controls (Table 4).

**Table 4:** Correlation between blood volume deviation, heart rate, and blood pressure

Dependent variable	Healthy male participants		Healthy female participants		Female patients with POTS	
	Pearson	*p*	Pearson	*p*	Pearson	*p*
Supine HR (bpm)	−0.212	0.486	−0.134	0.583	−0.566*	0.008
Supine SBP (mmHg)	−0.125	0.684	−0.056	0.819	−0.563*	0.008
Supine DBP (mmHg)	−0.395	0.182	−0.238	0.326	−0.387	0.083
Upright HR (bpm)	−0.387	0.191	−0.213	0.380	−0.608*	0.003

^*^p < 0.05; POTS, postural orthostatic tachycardia syndrome; HR, heart rate; SBP, systolic blood pressure; DBP, diastolic blood pressure

### Correlation with body impedance

**Fig. 5:**
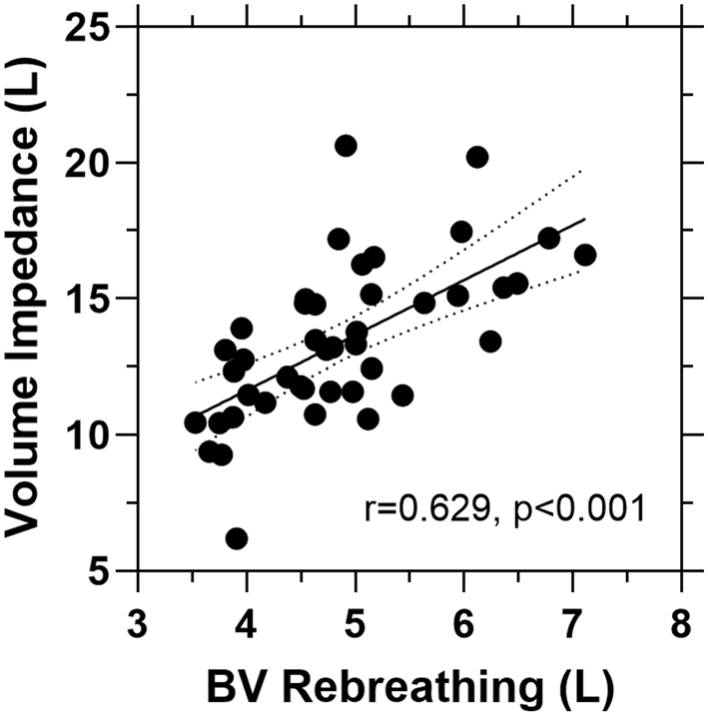
Correlation analysis between measured blood volume (BV) obtained from the carbon monoxide (CO) rebreathing method and total fluid volume estimated from body impedance

We measured the impedance of each body segment and calculated the median volume per body weight during 5 min of supine resting. Female patients with POTS had lower estimated fluid volumes than healthy female participants in the thorax, abdomen, and calf segments as well as the total volume across all segments. There were no significant differences in estimated volumes between healthy male and female participants (Table 5). The all-segment total fluid volume was also significantly and positively correlated with blood volume as measured by the CO rebreathing method (*r* = 0.629, *p* < 0.001; Fig. 5).

**Table 5:** Volume estimated by segmental body impedance in healthy participants and patients with postural orthostatic tachycardia syndrome (POTS)

Segment	Healthy male participants (*n* = 10)	Healthy female participants (*n* = 17)	Female patients with POTS (*n* = 20)
Thorax (mL/kg)	57.05 ± 19.78	56.61 ± 13.93	49.90 ± 14.57^*^
Abdomen (mL/kg)	71.55 ± 24.66	72.30 ± 15.39	61.08 ± 14.53^*^
Thigh (mL/kg)	27.10 ± 7.85	28.81 ± 7.71	26.53 ± 5.83
Calf (mL/kg)	8.12 ± 1.74	9.36 ± 1.79	7.94 ± 1.21^*^
Total volume (mL/kg)	199.03 ± 48.22	203.40 ± 32.61	182.86 ± 21.88^*^

Values presented as mean ± SD. * *p* < 0.05 between healthy female participants and female patients with POTS

## Discussion

We showed that the hemoglobin labeling technique by carbon monoxide was able to detect hypovolemia in patients with POTS on the basis of direct measurements of hemoglobin mass compared with estimates of predicted blood volume using the regression model of our control data. The blood volume deviation from the prediction was significantly associated with hemodynamic parameters including supine HR, upright HR, and supine SBP in patients with POTS (Table 4). We further show that blood volume estimated from semiautomated CO rebreathing and segmental body impedance were well correlated (Fig. 5). Several studies have reported that patients with POTS have lower blood volume than controls, using radioactive iodine [4, 25, 26] and dye dilution [4, 27]. Similarly, the semiautomated CO rebreathing method was sensitive to detect blood volume differences between patients with POTS and healthy female participants. Oberholzer et al. proposed a blood volume prediction formula constructed from the CO rebreathing method [17]. Their clinical equation requires lean body mass information, which was not available in our study. Thus, we performed our own regression model using data from our healthy controls. We found that POTS patients have a −13.92% blood volume deficit on average, which was clinically significant. On the other hand, Nadler’s formula underestimated normal volumes, resulting in nonphysiological positive BV_deviation_ in POTS with patients at +5.60%, indicating a hypervolemic status, which is unlikely. It is worth noting that Nadler’s formula was developed in the 1960s from radioactive (I^131^) labeled albumin blood volume data with weight, height, and sex as predictors [23]. The discrepancy between measured blood volume and Nadler’s predicted blood volume in our controls suggests that the blood volume predictive equation may be method specific.

We detected lower Hb_mass_ and RBCV in patients with POTS (Table 2), in agreement with previous studies [25, 28]. Raj et al. reported lower RBCV in patients with POTS when using a ^131^I-labeled human serum albumin dilution technique. The primary measurement of the ^131^I method is plasma volume, from which blood volume and RBCV are calculated indirectly using Hct [25]. The advantage of the CO rebreathing method is that it measures Hb_mass_ directly, independent of plasma and blood volumes (Eq. 1). Thus, the RBCV estimated by Hb_mass_ from the CO rebreathing method is arguably more accurate than that obtained from dilution techniques. Our results confirm the finding of low RBCV in patients with POTS. It has been proposed that patients with POTS have impaired renal production of erythropoietin and/or blunted renin counterregulatory mechanisms [25]. The higher Hct, despite lower Hb_mass_ and RBCV, in patients with POTS, compared with healthy female participants, suggests that the degree of plasma volume deficit is more severe than the RBCV deficit. Additionally, we could reproduce reported findings of healthy male participants having higher Hct, Hb concentration, and Hb_mass_ compared with healthy female participants [17, 29]. The differences in controls between female and male subjects emphasizes the important of sex-matched comparisons in POTS research, since most patients diagnosed with POTS are female [30].

Segmental body impedance showed lower fluid volume in patients with POTS compared with healthy female participants, corresponding to the CO rebreathing results (Table 5). The magnitude of the difference was greater in the abdominal region than the thigh and calf regions. This result explains the findings of previous study showing that use of a compression garment up to the abdominal level was more effective than the leg level in reducing orthostatic HR change [31]. The nonsignificant difference in the thigh region may result from a variation in the length of the thigh measurement, owing to limitations in approaching the groin region in female subjects. However, the impedance method yielded total fluid volume estimates that were nonphysiological and obviously higher than the blood volume measured by the CO rebreathing method (Fig. 5). Our impedance device uses alternating current at a frequency of 32.7 kHz, which is not specific for assessment of intravascular volume. The frequency used in impedance analysis varies among studies. Recommended frequencies < 5 kHz are used for extracellular fluid (ECF) and > 50 kHz for total body water (TBW) [18, 19, 32]. Probably, our device using a 32.7 kHz alternating current detects volumes beyond the ECF and certainly more than the intravascular volume. However, a positive correlation between the total volume estimated by the impedance and the CO rebreathing methods suggests that segmental body impedance could be useful in observing relative blood volume shifts, e.g., those induced by upright posture [20]. The impedance method used in this study was limited to a single alternating current frequency, which could not distinguish fluid in different compartments, e.g., extracellular and intracellular fluid. Future studies should apply multifrequency bioimpedance measurements to obtain more precise blood volume results.

A significant correlation between BV_deviation_ and resting heart rate, especially the upright heart rate, is consistent with the concept that hypovolemia is one of the pathophysiological mechanisms in POTS. The volume deficit did not cause low blood pressure, suggesting an intact baroreflex cardiac control and compensatory tachycardia [1]. Volume status did not correlate with either heart rate or blood pressure in controls, probably owing to the narrow physiological range of normal volumes. Besides baroreflex-mediated heart rate control, blood pressure can be maintained by adjusting cardiac contractility and peripheral vascular resistance. The significant correlation between blood volume and heart rate solely found in patients with POTS means that blood pressure control in patients with POTS is more dependent on the heart rate response than in healthy controls. Possible explanations of the heart-rate-dependent blood pressure control could be limited stroke volume changes due to cardiac deconditioning, or impaired vasoconstriction due to peripheral neuropathy in POTS [2, 5, 33]. Our findings of no difference in stroke volume but lower total peripheral resistance in patients with POTS support the possible impaired vasoconstriction. Unexpectedly, the blood volume in patients with POTS negatively correlated with baseline systolic blood pressure (Table 4). The cause could be overcompensation in patients with POTS. Sympathetic overactivity could lead to dehydration, which is described as catecholamine-induced blood volume contraction in patients with pheochromocytoma [34]. Further studies should be conducted to unravel the pathophysiology of POTS.

The CO rebreathing technique is a clinically feasible, noninvasive blood volume measurement. Subjects tolerated well the 10 min rebreathing through a mouthpiece in resting supine position [13]. The measurement can be performed within ~ 15 min after subjects remain supine to allow for proper fluid redistribution. The whole test and analysis can be done within a regular study room with a semiautomated rebreathing device, a gas analyzer, and a hematocrit device installed on a mobile cart. The semiautomated nature of the rebreathing device simplified the protocol and shortened the training time for new operators. These benefits of the method and the safety of being a radiation-free technique make the semiautomated CO rebreathing technique suitable for clinical practice.

## Limitations

The absolute blood volume values of the present study have not been confirmed by the gold-standard indicator dilution techniques, e.g., the chromium isotope-labeled red blood cell (^51^Cr-RBC) technique. Thus, the measurement errors of the CO rebreathing method could not be calculated. Also, we could not specifically determine absolute values of intravascular volume from the segmental body impedance. The impedance values were confounded by other fluid compartments such as intracellular fluid and intestinal fluid. Lastly, we cannot compare the volume status between healthy male patients and male patients with POTS owing to the low prevalence of POTS in male.

## Conclusions

We present noninvasive blood volume measurements using a novel semiautomated CO rebreathing method and regional fluid estimation by segmental body impedance method in patients with POTS and healthy female participants. The semiautomated CO rebreathing method was able to detect differences between patients with POTS and healthy participants, including lower Hb_mass_, red blood cell volume, plasma volume, and blood volume in patients with POTS. Although segmental body impedance overestimated fluid volume beyond physiological ranges, it could still detect the volume differences between patients with POTS and healthy female participants. The all-segment total fluid volume computed from segmental body impedance positively correlated with the CO rebreathing blood volume, suggesting that the impedance method could be used for relative volume changes. We found a significant negative correlation between blood volume status and heart rate in patients with POTS but not in controls, suggesting that blood pressure control is more dependent on heart rate in patients with POTS than in healthy subjects. Accurate volume estimation by the semiautomated CO rebreathing method provides valuable information that can be useful in the interpretation of autonomic function tests and optimization of therapy for patients with POTS.

## Data Availability

Data are available upon request.
